# A Histological Comparison of a New Pulp Capping Material and Mineral Trioxide Aggregate in Rat Molars

**Published:** 2013-12-24

**Authors:** Fariborz Moazzami, Yasmin Ghahramani, Ali Mohammad Tamaddon, Ali Dehghani Nazhavani, Alireza Adl

**Affiliations:** a *Department of Endodontics, Dental school, Shiraz University of Medical Sciences, Shiraz, Iran; *; b *Postgraduate Student, Department of Endodontics, Dental School Student Research Committee, Shiraz University of Medical Sciences, Shiraz, Iran;*; c *Center for pharmaceutical Nanotechnology and School of Pharmacy, Department of Pharmaceutics, Shiraz University of Medical Sciences, Shiraz, Iran;*; d *Department of Oral and Maxillofacial Pathology, Dental school, Shiraz University of Medical Sciences, Shiraz , Iran*

**Keywords:** Cell Differentiation, Dental Pulp Capping, Dexamethasone, Inflammation, Mineral Trioxide Aggregate, MTA, Odontoblast, Vitamin D

## Abstract

**Introduction**: Recent investigations have attempted to improve regenerative endodontics with the help of stem cell therapy. *In vitro* studies have shown the ability of different agents to stimulate the differentiation of dental pulp stem cells (DPSC) into odontoblast-like cells. A combination of dexamethasone, β-glycerophosphate and Vitamin D has been proven to induce a successful differentiation. The aim of this animal study was to evaluate the effect of this combination, named odontoblastic differentiating material (ODM), on pulp tissue when used as a capping material.** Materials and Methods:** Sixty maxillary right and left molars of 30 Sprague-dawley rats were selected for this study. The teeth were exposed under sterile condition. Half of the teeth were capped with mineral trioxide aggregate (MTA) and the other half with ODM. All cavities were restored with glass ionomer. The rats were sacrificed at post-operative intervals of 2 weeks and 2 months. Samples were histologically evaluated for the degree of inflammation and reparative dentin formation. Finally the data was analyzed with Mann-Whitney and Chi-Square tests. **Results:** Reparative dentin formed in all groups within both time periods and there was no statistically significant difference between the groups in the mentioned time periods. The MTA group, however, showed a statistically significant reduction in inflammation at both time intervals (*P<*0.05). Compared to MTA, ODM samples showed a greater amount of inflammation in the pulp tissue. Conclusion: ODM, as a pulp capping material, can induce dentinal bridge formation.

## Introduction

Regeneration therapy may revolutionize the future of endodontic. Following biological procedures, the goals of regenerative endodontics are replacing damaged or diseased tissue and maintaining the physiological function of pulp-dentin complex and root structures [1]. Regarding this concept, regenerative endodontics include: direct pulp capping, revascularization, apexogenesis, stem cell therapy and tissue engineering [2, 3]. Undoubtedly, dental stem cells play pivotal role in regenerative endodontics. There are five types of stem cells which can potentially differentiate into odontoblast-like cells [1]: 1) dental pulp stem cells (DPSC) [4], 2) stem cells of apical papilla (SCAP) [5], 3) dental follicle progenitor cells (DFPC) [6], 4) human exfoliated deciduous teeth (SHED) [7] and 5)bone marrow mesenchymal stem cells (BMMSC) [8].

DPSC are highly proliferative, self renewal cells which are located in the prevascular niche and the cell rich zone of Hohl near the odontoblastic layer [9, 10]. Growth factors are necessary for these cells to differentiate into odontoblast-like cells. There are different types of growth factors which can play a role in regeneration.

Recent studies showed an intensive increase in differentiating human dental pulp cells into odontoblast-like cells via administrating dexamethasone (Dex) to the culture media. This effect was more prominent when 1, 25-dihydroxyvitamin D3 (Vit D3) was combined with Dex [11, 12]. A novel *in vitro* study revealed that Dex can induce a 2-fold increase in alkaline phosphatase (ALP) activity after 7 days and 350% increase after 14 days, deciphering odontoblastic differentiation [13].

Another odontoblastic differentiation inducing agent is β-glycerophosphate (β–Gly). The degree of differentiating DPSC into odontoblast is depends on the concentration β-Gly; with the best concentration for differentiation and maturation of odontoblasts being 5 mM [14]. Couble *et al.* also showed that the addition of β–Gly to the culture medium of dental pulp cells induces odontoblastic features [15].

To generate odontoblasts from cultured dental pulp cells, Tonomura *et al*. showed significant ALP activity by adding a combination of Dex, β–Gly and Vit D3 to dental pulp cell culture media [16]. Another *in vitro* study on cultured rat dental pulp cells, showed that Dex, β–Gly and L-ascorbic acid can differentiate rat DPSC into odontoblast-like cells and produce calcified nodules within 3 weeks; in a similar study on human DPSC *in vitro* nodule formation occurred during 4 weeks [17, 18].

As mentioned earlier, odontoblastic differentiating materials were used as an additive to DPSC culture media in some *in vitro *studies. The goal of this study is to administer the combination of odontoblastic differentiating materials (Dex, β–Gly and Vit D3) sequestered in a polymeric vehicle, as a direct pulp capping material in an animal study model. To see the effect of this combination *in vivo*, optimum concentration of all agents were carried into a biocompatible vehicle which could release the agents gradually and continuously. This new pulp capping agent is named as odontoblastic differentiating material (ODM).

## Material and Methods

***DOM preparation***

A combination of active ingredients *i.e.* 1, 25-dihydroxy vitamin D3 (Iran Hormone Co., Tehran, Iran), β-glycerophosphate disodium salt hydrate (Sigma Aldrich, St. Louis, Missouri, USA) and dexamethasone (Sigma Aldrich, St. Louis, MO, USA) were triturated in a clean mortar with a pestle. The required amount of the powder mixture was weighed with a precision balance (Ohaus Scale Medic GT 210, Florham Park, NJ, USA) and wetted with a few drops of Tween 20 (Sigma Aldrich, St Louis, MO, USA). Then, it was dissolved in normal saline with equal volume shaken vigorously followed by probe ultrasonication (Hielscher, UP200H, 24 kHz, Germany) for 30 sec. In parallel, a polymer blend containing sodium carboxymethylcellulose (Sigma Aldrich, St Louis, MO, USA), hydroxypropyl methylcellulose (viscosity 40-60 cP, Sigma Aldrich, St Louis, MO, USA) and carbopol 934 at respective weight ratio of 3:1:1 were mixed by spatulating on a glass slab. The blend was transformed into a sticky paste by dropwise adding of an exact volume of the pre-dispersed active ingredients in normal saline. The product was used as a direct pulp capping material in rat molars.

***Operative procedures***

Fifteen male and fifteen female Sprague-dawley rats (providing 60 molars) with an average age of 3 months and body weight between 220 and 250 g were selected for this study. The protocol of the study was approved by the ethics committee of Animal Research Center of Shiraz University of Medical Sciences. The animal model was chosen in accordance with protocol for pulp capping for new material evaluation before using on human to minimize atypical response [19].

The rats were anesthetized by a combination of 1 mg/kg ketamine hydrochloride (Alfasan International, Woerden, the Netherlands) and 1.5 mg/kg xylazine (Alfasan International, Woerden, the Netherlands) intramuscular injection. Before cavity preparation, caries free maxillary first molars were disinfected with 0.2% chlorhexidine gluconate (Shahrdaru, Tehran, Iran). Carl Zeiss surgical microscope (OPMI PRO magis, Carl Zeiss, Oberkochen, Germany) was used during operative procedure.

An occlusal cavity was prepared on the right and left maxillary first molars of each rat with a high speed handpiece with water spray and a cylindrical diamond bur (model 801, size 012, Komet, Gebr. Brasseler, Lemgo, Germany). The roof of the pulp chambers were perforated with a sharp probe. Having controlled pulpal bleeding, remnant blood was removed from the cavity by sterile paper points. Right maxillary first molars were directly capped with ODM and left maxillary first molars with mineral trioxide aggregate (ProRoot MTA, Dentsply, Tulsa Dental, Tulsa, OK, USA); this application closely matched the manufacturer’s specification (1 g MTA mixed with 0.35 g water). All cavities were then filled with light-cured restorative glass ionomer (GC International crop, Tokyo, Japan).

To evaluate the effect of polymeric vehicle of DOM and restoration material, as control groups, 10 maxillary right molars were capped directly with DOM vehicle and 10 maxillary left molars with light-cured restorative glass ionomer, and then both were restored with light-cured restorative glass ionomer. After the operation, the rats were supervised according to the protocol of Animal Research Center of Shiraz University of Medical Sciences, with a special soft diet and supporting analgesics.

**Figure 1:**
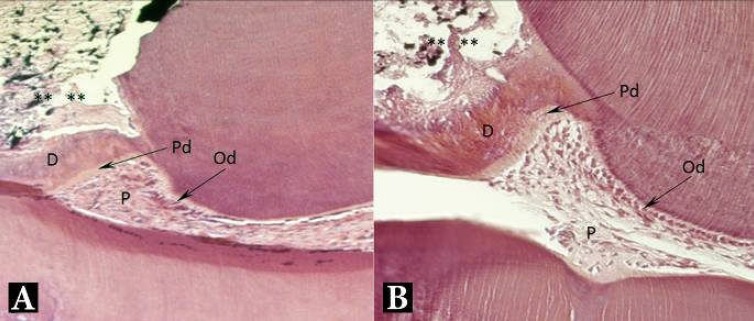
Light microscopy micrograph of a maxillary rat molar treated with: *A)* MTA and *B)* ODM after 2 weeks of interval. Formation of dentin bridge at pulp-capping material interface. (**) exposure area, (D) dentin bridge, (Pd) predentin, (Od) odontoblasts, (P) pulp. H&E, 200×

**Figure 2:**
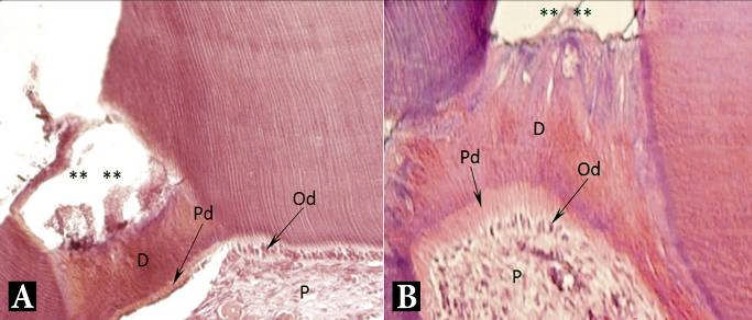
Light microscopy micrograph of a maxillary rat molar treated with: *A)* MTA and *B)* ODM after 2 months. Formation of dentinal bridge at the pulp-material interface. Predentin and well organized odontoblastic layer are prominent. (**) exposure area, (D) dentin bridge, (Pd) predentin, (Od) odontoblasts, (P) pulp. H&E, 200×

**Figure 3:**
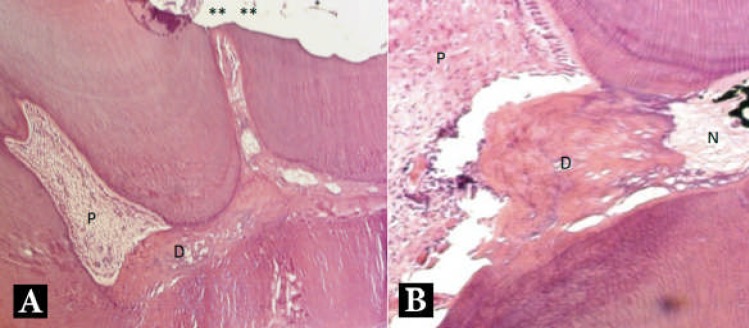
*A)* Light microscopy micrograph (100×) of a maxillary rat molar treated with MTA after 2 months. Reparative dentin formation is detectable on the floor and roof of the pulp chamber in contact with one another. Lacunas are also detectable; *B)* Light micrograph (200×) of a maxillary rat molar treated with ODM after 2 months of interval. A dentin bridge with lacuna separates the necrotic area from the vital pulp. (**) exposure area, (D) dentin bridge, (P) pulp, (N) necrotic area. H&E

Rats were sacrificed by carbon dioxide inhalation at post operation intervals of two and eight weeks. The maxilla of the rats were placed in 10% buffered formalin solution for 2 weeks, and then were demineralized for 4 weeks in 10% formic acid. The specimens were embedded in paraffin after removing the restorative materials and 5 µ thickness serial sections were prepared through the exposure site and were stained with hematoxylin and eosin (H&E). Histological evaluation was performed by an oral and maxillofacial pathologist using a high power field optical microscope as follows:

*I)* the coronal pulp was histologically examined for pulpal inflammation including examination for bacterial infection and presence of inflammatory cells. The degree of inflammation was classified in groups 0 to 4 as follows:

0-Normal pulp structure

1-Mild: an increase in fibroblast, capillary and inflammatory cells and few extravasated red blood cells 

2-Moderate: characterized by more inflammatory cells and increasing capillary and vessels

3-Severe: significant cellular infiltration, excessive blood vessels

4-Necrosis of the pulp [20]

*II)* The pulp was also examined for differentiation of DPSCs into odontoblasts or odontoblast-like cells.

*III)* The thickness of reparative dentin, more specifically the dentinal bridge beneath the capping area, was measured in the pulp chamber.

The data were analyzed with the Kruskal-Wallis and the Mann-Whitney tests. Statistically significant differences were set at *P*<0.05.

## Results

***Histopathologic evaluation of the pulp after two weeks***

Table 1 shows the frequency distribution of pulpal Inflammation in 2 weeks and 2 months Specimens. The difference in pulpal inflammation between MTA and ODM samples was statistically significant within 2-week interval (*P*=0.001) (Table 1).

All the specimens in the MTA group were vital, although some degrees of hyperemia were detected in the pulp of half of the specimens. Mild inflammation (grade I) was observed in 20% of MTA group while 20% of the samples showed moderate to severe inflammation (grade III) accompanied with chronic inflammatory cells.

Compared to the MTA group, although most of the specimens (80%) were vital, inflammation in the ODM group was more prominent. Accumulation of chronic inflammatory cells beneath the capping area (grade III) was observed in 50% while mild inflammation (grade I) was observed in 20% of specimens. Despite the presence of different degrees of inflammation in both groups, odontoblastic differentiation and reparative dentin formation were notably detected in both groups (Figure 1). However, there was no significant difference between the two groups within 2 weeks (*P*=0.6) (Table 2).

In the control groups, the specimens were necrotic in both groups; whether the one treated with the vehicle of ODM (without active ingredient) and the group capped with glass ionomer. Neither odontoblastic differentiation nor reparative dentin formation was detected in the control groups. 

***Histopathologic evaluation of the pulp after two months***

The difference between pulpal inflammation in MTA and ODM treated teeth was statistically significant (*P*=0.028) (Table 2).

Odontoblastic differentiation and reparative dentin formation occurred in both groups (Figure 2), but statistical analysis did not reveal statistically significant difference (*P*>0.847) (Table 2). Despite the similar statistical results in reparative dentin formation, maximum mean thickness of the reparative dentin formed belongs to the ODM treated teeth, about 100 µm.

**Table 1:** Frequency distribution of pulpal Inflammation in two-week and two-month Specimens

**Inflammation degree**	**Two weeks**		**Two months**	
	**MTA**	**ODM**	**MTA**	**ODM**
**0**	1	0	1	0
**1**	4	0	5	0
**2**	2	3	0	0
**3**	3	5	2	8
**4**	0	2	2	2

**Table 2:** Mean (SD), minimum and maximum of reparative dentin thickness of specimens in two-week and two-month intervals (*n*=10)

	**Two weeks**		**Two months**	
	**MTA**	**ODM**	**MTA**	**ODM**
**Mean (SD)**	30 (8.4)	15 (14)	45.2 (20.2)	45 (25)
**Min** **thickness**	17	0	24	25
**Max** **thickness**	43	33	82	100

In both groups of two-month specimens, in addition to the formation of dentinal bridge under the capping area, reparative dentin formation was observed in the pulp horns, floor and walls of the pulp chamber. Under the capping area, a well organized tubular dentin bridge with predentin and odontoblastic layer was observed while in the pulp horns and on the floor of the pulp chambers the reparative dentin had an atubular structure containing lacunas. This atubular dentin was mostly detected in the MTA group (Figure 3). Although most of the pulps in the ODM group were vital, those with reparative dentin formation showed a partial necrosis beneath the capping area and the reparative dentin, as a noticeable barrier separating the necrotic zone from the vital pulp (Figure 4).

All the specimens were necrotic in both control groups; no signs of odontoblastic differentiation or reparative dentinogenesis were detected.

## Discussion

To the best of our knowledge, there was no study evaluating the combination of odontoblastic differentiating materials (ODM) as a pulp capping material in an *in vivo* model. On the basis of the previous *in vitro* studies, showing the positive effect of ODM on DPSC differentiation [11-13], the null hypotheses of the present study was to determine if ODM can induce odontoblastic differentiation and dentin bridge formation in an *in vivo* scenario.

Direct pulp capping is a type of vital pulp therapy procedure in which sealing the exposed pulp with biocompatible, antibacterial dressing is necessary [21]. Ease of handling, sealing ability, marginal adaptation, long term stability and stimulation of calcified bridge are also important in dressing characteristics [22, 23]. A pilot study showed ODM sets in thirty min however, further investigation is needed to define the exact characteristics of ODM.

To evaluate the pulp healing after direct pulp capping procedure, in many regenerative endodontic studies, rat molars have been used mainly because of their availability, inexpensiveness to purchase and maintenance as well as their histological and physiological similarities to human pulp [24, 25].

In the present study, 2 week- and 2 month- intervals were selected to evaluate the effect of capping materials. The same periods were used in several other studies evaluating pulp responses to different pulp capping materials [26-28]. 

In different studies, MTA is the material of choice for direct pulp capping because of its biocompatibility, sealing ability and quality of dentin bridge formation. Therefore, in the present study, MTA was chosen as the gold standard to be compared with ODM as a pulp capping material.

According to the histological results, 2 weeks after direct pulp capping with MTA and ODM, dentin bridge was detectable in both groups and there was no statistical significant difference between them. This showed that ODM also could induce reparative dentin formation in the *in vivo* situation, approving the *in vitro* studies in which this combination induced odontoblastic differentiation and dentin formation. Like other studies, in the present study, MTA showed the ability to form dentinal bridge in a two-week interval.

In both groups of the two-month specimens, a well organized odontoblast-like cell layer, predentin and reparative dentin were detected which can be interpreted as a sign of healing and a good response to pulp capping materials. Although there wasn’t a significant difference between MTA and ODM groups with regard to mean thickness of the reparative dentin formed within 2 months, the maximum thickness of the formed bridge happened in the ODM group. This may show the continuous activity of the ODM ingredients resulting in a thicker formed bridge.

Both control groups, either treated with the vehicle of ODM or glass ionomer, did not form calcified bridges in any intervals. This shows the ability of ODM and MTA in odontoblastic differentiation and dentin bridge formation.

According to our study, there were no noticeable differences in the location and continuity of the dentinal bridge between ODM and MTA groups, confirming previous studies about MTA ability in dentin bridge formation [29-31]. The pulpal inflammatory reaction was evaluated by the amount of inflammatory cells invaded beneath the capping area, increasing in capillaries, blood vessels and hyperemia: these criteria show pulpal vitality status and biocompatibilities of the capping material.

Specimens capped with ODM showed identical signs of inflammation in both time periods: the invasion of chronic inflammatory cells beneath the capping area and increase in the number of capillaries. In this study, in both intervals, MTA showed lower levels of inflammation compared to the ODM group. In other studies, comparing MTA to the pulp capping materials like calcium hydroxide and propolis, the least inflammation was also reported for MTA [32, 33].The higher inflammation of the ODM group might be attributed to its consistency and handling. It is worth mentioning that even through microscope magnification, the application of a sticky white paste into the occlusal cavity of the rat molars was complicated and might have affected the sealing quality of ODM group contributing to higher inflammation comparing to MTA specimens. The other reason over the presence of inflammatory cells in the ODM specimens is the type of the vehicle used for loading the active ingredients contributing to inflammation in this group.

In the control group, samples treated with ODM vehicle showed complete necrosis of the pulp tissue with no signs of barrier formation in both time periods. Therefore, it is assumed that if ODM is applied with a different vehicle, better result might be achieved.

## Conclusion

In conclusion, although ODM as a capping material has the ability to stimulate the pulp to form a dentinal barrier, it tends to induce more inflammation in the pulp tissue when compared with MTA. Further investigations are needed to find a proper vehicle for ODM that will improve its consistency and biocompatibility.
